# Distal and Proximal Factors of Health Behaviors and Their Associations with Health in Children and Adolescents

**DOI:** 10.3390/ijerph10072944

**Published:** 2013-07-16

**Authors:** Lena Lämmle, Alexander Woll, Gert B. M. Mensink, Klaus Bös

**Affiliations:** 1 Technische Universität München, Georg-Brauchle-Ring 60/62, München 80992, Germany; 2 Karlsruhe Institute of Technology, Engler-Bunte-Ring 15 Geb. 40.40, Karlsruhe 76131, Germany; E-Mails: Alexander.Woll@kit.edu (A.W.); Klaus.Boes@kit.edu (K.B.); 3 Robert Koch Institute Berlin, Postfach 650261, Berlin 13302, Germany; E-Mail: MensinkG@rki.de

**Keywords:** psychosocial determinants, health behavior, objective health, health complaints, subjective health

## Abstract

*Objective*: The aim of the present paper was to analyze factors affecting distal and proximal health behavior within a biopsychosocial model for examining their interactions and associations with respect to health. *Methods*: Path analysis was based on the nationwide, cross-sectional German Health Interview and Examination Survey for Children and Adolescents (2003 to 2006). The data was collected from 4,529 participants with an average age of 9.45 years (*SD* = 4.01). Socio-demographic data, psychosocial factors and health behavior were assessed via questionnaire. Participants also underwent physical fitness tests and a medical examination. *Results*: Over the five levels of the model analyzed with socioeconomic status, immigration background, and rural-urban differences on the first level; physical activity of relatives and peers, intrinsic motivation, and quality of life on the second level; eating patterns, sedentary behavior, and physical activity on the third level; physical fitness and objective health on the fourth level; and health complaints and subjective health on the fifth level; direct, moderation, and mediation effects could be shown. *Conclusions*: Several distal and proximal factors are needed to take account of the multivariate complexity of health: e.g., immigration background affected health behaviors only indirectly and the effect of physical activity on objective health was mediated by physical fitness.

## 1. Introduction

The value of physical activity (PA) [1,2], appropriate sedentary behavior (SB) [2,3] and appropriate eating patterns [4,5] on the healthy growth and development of children and adolescents is well known. Furthermore, active and energetic children tend to remain active throughout middle and late adulthood [6], whereas sedentary children are more likely to become sedentary adults [3]. Aside from PA and SB, eating patterns are behavioral patterns that are also often carried out into adulthood [4]. Nevertheless, there has been evidence observed for decreasing levels of PA [7], for a trend towards SB [2], and for a decline in the quality of eating patterns [4]. Because changing these behaviors requires considerable conscious effort [8], the research focus should be on prevention rather than treatment [9]. Risk factors for unhealthy stable behaviors and their consequences should therefore be established [10]. In the present study, we consequently considered risk factors of health-relevant behavioral patterns in children and adolescents and the associations of these patterns with objective health within a biopsychosocial model. This model is described in the following paragraph. 

### 1.1. Relevance of Biopsychosocial Approaches

To increase the ability to predict and potentially prevent health risk behaviors, and consequently objective health, both distal and proximal determinants of those behaviors should be considered. Distal determinants tend to be more stable relative to proximal determinants and are further expected to influence health behavior mainly via these proximal determinants [11,12]. Consequently, it has been increasingly recognized that there is a need to integrate theories in order to prevent the development of complex health risk-behaviors [13]. Existing comprehensive theories connected to health behavior are, for example, the theory of triadic influence [11] and the Integrated Change Model [14]. Accordingly, empirical research has not only observed associations within distal and proximal determinants [15,16], but also effects of distal and proximal determinants on health behaviors [17,18]. Furthermore, associations within health behaviors and associations between health behaviors and objective health have been observed [19]. However, such empirical evidence is mostly based on bivariate findings and not on comprehensive approaches, as is suggested by theory [11,12,13,14]. Thus, a comprehensive, distal-proximal framework is needed to examine if support for the assumed complex interplay of distal and proximal factors on health behavior and objective health can be provided. This also enables one to examine if recent bivariate findings still hold true when considering them comprehensively: more precisely, it will be possible to examine if there are moderation, mediation suppression or effects of common variance between distal factors, proximal factors, health behaviors and objective health. As this is a first attempt at combining several recent bivariate findings comprehensively, no concrete assumptions are made on how these effects will be precisely manifested. Rather, the focus was on exploring if and how these effects will manifest themselves (generating hypotheses). Finally, this information can function as an aid in identifying the level—distal or proximal—on which interventions would be most fruitful. 

**Figure 1:**
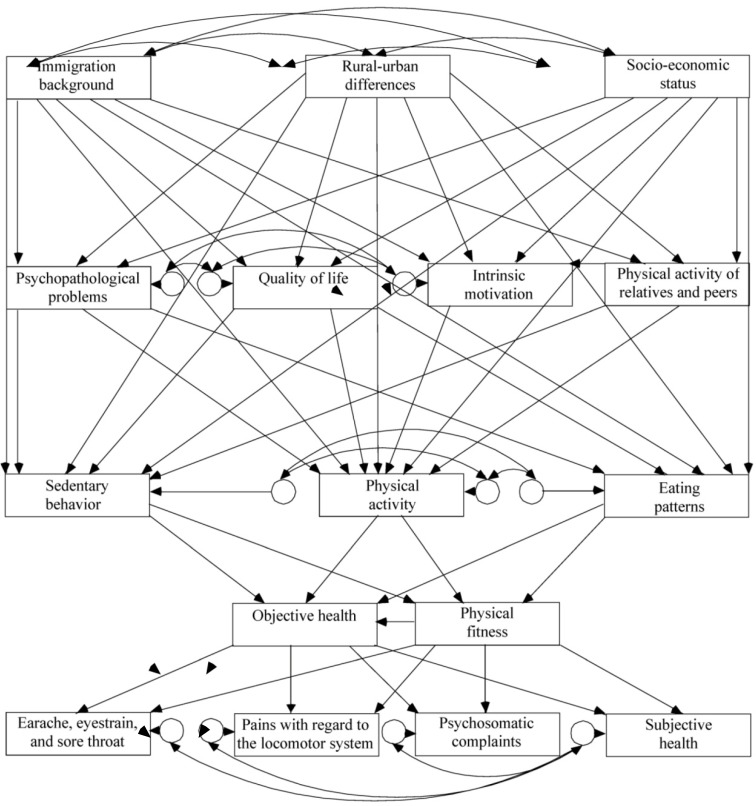
Biopsychosocial model.

The biopsychosocial model considered here therefore simultaneously examines distal factors on the first level (operationalized with the environmental factors of socio-economic status (SES), rural-urban differences and immigration background; see Section 4.1.1), proximal factors on the second level (operationalized with the more individual—interpersonal as well as intrapersonal—factors of physical activity of relatives and peers (interpersonal factor), intrinsic motivation, psychopathological problems and quality of life (intrapersonal factors) and behavioral factors (third level; operationalized with PA, SB and eating pattern) on physical fitness and objective health (fourth level consisting of objective physical measures) and on subjective health and health complaints (fifth level) [20]. It is assumed that the environment influences individual factors and that both result in health behaviors. Health behaviors are then assumed to affect body composition, which leads to subjective health and health complaints. Within each level, associations (e.g., between behaviors, PF as a predictor of objective health) are possible, but not mandatory. Figure 1 illustrates the basic idea of the model. For operationalization information see method section. 

### 1.2. Objectives of the Present Study

With the assistance of the biopsychosocial model, the objectives of the current paper were to provide evidence for comprehensive approaches and therefore to analyze several causes—distal as well as proximal—for disparities in PA, SB, and eating patterns among German children and adolescents, and their relationships with objective health, subjective health and health complaints. This concept is a first attempt to examine a significant number of correlates, both those that are well-known (e.g., correlations between SB and premature morbidity [21]; for an overview of these past bivariate correlations see Section 4.1.1) and those that are hardly known at all, from a biopsychosocial perspective, but for which research has been demanded (e.g., correlations between rural-urban differences and SB [19]). It therefore also offers the opportunity to examine whether past bivariate correlations still hold when viewed more comprehensively. 

## 2. Method Section

### 2.1. Participant Characteristics

The following analyses were based on the German Health Interview and Examination Survey for Children and Adolescents (KiGGS), which was conducted between May 2003 and May 2006. The KiGGS survey is a nationwide, cross-sectional study on the health status of children and adolescents between the ages of 0 to 17 years, which was conducted by the Robert Koch Institute of Berlin (RKI) [22]. The KiGGS survey was complemented by the “Motorik”-Module (MoMo) for a more differentiated recording of physical activity and physical fitness in children and adolescents between the ages of 4 and 17 years [23]. Data was collected from 4,529 children and adolescents (2,244 girls and 2,285 boys) between the ages of 4 and 17 with an average age of 9.45 years (*SD* = 4.01). 

### 2.2. Sampling Procedures

Participation was voluntary. Participants were chosen nationwide with the use of a stratified, two-stage probability sampling procedure. First, a systematic sample of 167 primary sample units was drawn from an inventory of German communities stratified according to the BIK classification system, which measures the grade of urbanization, and the geographic distribution. The number of primary sample units per strata was determined using the Cox procedure for community sampling with a sampling probability proportional to population size [24]. Second, an equal number of addresses per birth cohort were randomly selected from local population registries within selected primary sample units. Finally, a random sample was drawn, including a total of 8 to 10 children and adolescents per birth cohort, depending on community size. Invitations were sent to their home addresses. Participants were invited to local study centers to fill out questionnaires and take part in the study. The response rate was 66.6%, with 5.3% quality-neutral drop-outs and with low variation with regard to the age groups [25]. In order to reach a high response rate, a strong focus was put on public relations work. Participants were informed about the examination. Not only professional flyers and postcards, but a service telephone was also provided in addition to the professional flyers and postcards. Local and supraregional media were informed one week before the invitation of the participants. The integration of local institutions like health departments and schools were also part of the public relations work [26]. The study was carried out according to the ethical guidelines laid down by the medical ethics committee of Charité University Clinic at the Humboldt University of Berlin. 

### 2.3. Sample Size

A total of 28,899 children and adolescents were randomly selected and invited to participate in the KiGGS study, 1,512 (5.3%) were later classified as quality-neutral drop-outs. For the MoMo substudy, only 4586 families with children between the ages of 4 and 17 were randomly selected from the KiGGS study and invited to participate. A total of 98.6% (*n* = 4,529) of those who agreed to participate, actually took part [23]. All measures used in the biopsychosocial model were chosen from the KiGGS experts (amongst others, medical physicians, psychologists, nutritionists, sports scientists) with respect to previous theoretical assumptions and empirical findings in international child and adolescent health research (see Section 4.1.1). 

### 2.4. Measures

#### 2.4.1. Immigration Background

Participants were considered as children and adolescents with parents with migration background (coded as 1) if they had emigrated from another country and if at least one parent was not born in Germany, or if both parents had emigrated from another country and had not accepted the German nationality. All other participants were treated as non-immigrants (coded as 2). Information on citizenship, country of birth, year of entry, and age at entry was assessed using a questionnaire. 

#### 2.4.2. SES

The SES information was based on the mean score of parents’ information concerning their school education (What is your high-school graduation level? Please mention only the highest grade) ranging from 1 *(without graduation*) to 7 (*high-school diploma*); professional qualification (Have you completed a university education? If yes, in what field?) ranging from 1 (*without professional qualification*) to 7 (*university degree*); occupation (What is your current main professional position?) ranging from 1 (*common laborer*) to 7 (*leading position*); and the net household income (How high is the average monthly household income, that is, the net household income of all members of the household after payroll and social security contributions?) ranging from 1 (*<1*,*250 euros*) to 7 (*>5*,*000 euros*). The mean score ranged from 1 (*low SES*) to 7 (*high SES);* Cronbach’s alpha = 0.69. The parent with the higher status, or, in the event of a divorce, the status of the parent where the child was living, was chosen [27]. 

#### 2.4.3. Rural-Urban Differences

Rural areas (coded as 1) were defined as rural (population value: <5,000) and provincial areas (population value: 5,000–20,000), whereas urban areas (coded as 2) were defined as medium-sized towns (population value: 20,000–100,000) and metropolitan areas (coded as 3; population value: >100,000) [23].

#### 2.4.4. Intrinsic Motivation

According to the Theory of Reasoned Action, attitude, and, as a follow-up, intention towards PA are important determinants of PA levels. Attitude is a function of the belief that participation in PA will result in certain outcomes [28]. Such outcome expectations include fun, social aspects, and skill development [29]. Therefore, nine statements such as “I participate in sports in order to have something to do with my friends” and “I participate in sports in order to have fun” were used and evaluated with the aid of a 5-point Likert scale, ranging from 1 (*I strongly disagree*) to 5 (*I strongly agree*; Cronbach’s alpha = 0.79). Finally, a mean parcel over these items has been built. 

#### 2.4.5. QOL

To assess QOL, the KINDL-R questionnaire was used. The psychometric properties of the KINDL-R revealed a high degree of reliability and the procedure revealed a satisfactory convergent validity for the present data [30]. Cronbach’s alpha is 0.85. 

#### 2.4.6. Psychopathological Problems

To assess psychopathological problems, the strengths and difficulties questionnaire (SDQ) was used. The psychometric properties of this screening instrument have been shown to be satisfactory to good for the present data [31]. Cronbach’s alpha in the present data is 0.80.

#### 2.4.7. PA (of Relatives and Peers)

PA was assessed using the MoMo PA questionnaire containing previously validated items involving the duration, intensity, and frequency of PA in leisure time, school, and sports clubs in the past year. It consists of 35 items and has been developed in orientation to existing questionnaires. The test-retest reliability in a previous 7-day longitudinal study ranged from *r_tt_* = 0.72 (PA in leisure time) to *r_tt_* = 0.93 (PA in sports clubs). Construct validity related evidence was gathered by looking at the correlation with a multisensory electronic monitor including a biaxial accelerometer measuring PA (SenseWear Pro 2) [23]. Results have shown higher correlations (*r*_min_ = 0.56, *r*_max_ = 0.66) in comparison with other PA questionnaires [32].

In accordance with international recommendations [33,34] for activity guidelines with at least 60 min of moderate to vigorous activities daily, a PA parcel was built. For school, leisure time, and sports club activity, only time spent from at least moderate PA in min was considered: number of school sport hours × 30 min + frequency of first sport × duration of first sport × number of months first sport is undertaken/12 (months a year) + frequency of second sport × duration of second sport × number of months second sport is undertaken/12 (months a year) + frequency of third sport × duration of third sport × number of months third sport is undertaken/12 (month a year) + frequency of fourth sport × duration of fourth sport × number of months fourth sport is undertaken/12 (months a year). Sustained moderate to vigorous PA was documented as being associated with positive health outcomes [35]. This questionnaire also included information about the PA patterns of relatives and peers. Children and adolescents were asked if their parents, siblings, and peers exercise regularly (yes/no).

#### 2.4.8. Eating Patterns

For the derivation of eating patterns, information from a semi-quantitative food frequency questionnaire (FFQ) was used [36]. The design of the FFQ was inspired by the US National Institute of Health’s Diet History Questionnaire [37]. The FFQ has been validated for adolescents [36]. In addition, in a pilot study among parents of younger children and adolescents, the questionnaire was tested, and included a cognitive debriefing. In conclusion, it was very well understood by the respondents, and the duration of completion was acceptable (about 15–20 min). The development of the FFQ is described in detail elsewhere [36]. The FFQ retrospectively assesses the frequency of consumption and average portion amounts of 45 food groups during the “last few weeks”. In the present analysis, only 22 food items were used. These items employed could be clearly assigned to either healthy or unhealthy eating, such as fruit and vegetable consumption, sugar-sweetened beverage consumption, or sugar snacking [38]. After recording the unhealthy eating pattern, the amount of food consumption was calculated by multiplying the frequency and portions and then summing these products. Cronbach’s alpha for the present data is = 0.63.

#### 2.4.9. SB

The average time of television viewing and playing computer games on weekdays and on weekends/holidays was assessed to describe SB. The 5-point scale ranged from 1 (*not at all*) to 5 (*more than 4 h a day*; Cronbach’s alpha = 0.70). Questions were assessed using the Health Behavior in School-aged Children Questionnaire of the World Health Organization [23]. For parceling procedure, a mean score was calculated. 

#### 2.4.10. PF

Participants were tested with eight tests of the “Motorik”-Module (MoMo) test battery [39] to assess a complete PF profile involving endurance (through a bicycle ergometer test), strength (through push-ups, standing long jump, and force plate for high jumps), coordination under precision demands (through standing on one leg and balancing backwards), coordination under time pressure (through jumping sideways), and flexibility (through forward bending of the trunk). The content-related validity of all tests was evaluated as good (*M*_Significance _ = 1.9, *M*_Practicability_ = 1.7) throughout with regard to significance and feasibility as based upon expert ratings. Furthermore, good test-retest reliability coefficients (*r*_min_ = 0.74, *r*_max_ = 0.96) were found. The retest was conducted four days after the first test [23]. All measures were z-transformed and a mean score was calculated. 

#### 2.4.11. Objective Health Parameters

Body mass index (BMI), skinfold thickness, blood pressure, and cholesterol were assessed. Body height (stature) was measured while standing with a calibrated, portable Holtain stadiometer and an accuracy of up to 0.1 cm. Participants were measured for body weight (mass) in their underwear on a calibrated scale with an accuracy of up to 0.1 kg. BMI (body weight (kg)/height (m^2^)) was calculated as an index of body fat. Because BMI shows the excessive increase of fatty tissue and its distribution only insufficiently, skinfold thickness (SFT) was assessed for estimating body fat with the aid of a Holtain caliper using two measurement points on the right side: triceps (the back of the upper arm) and subscapula (beneath the edge of the shoulder blade). Blood pressure and heart rate were assessed twice on the right side with the elbow at heart level. Systolic, diastolic, and arterial medium pressures, as well as pulse rate, were determined with the use of an automatic hemodynamometer (Datascope Accutor Plus). The first automatic measurement was taken while the participant was sitting after a 5 min break and the second measurement was taken after an eye test. The size of the Datascope blood pressure cuff was chosen with regard to the requirement of covering at least two thirds of the upper arm above the brachial artery. Cholesterol is described by HDL-cholesterol and LDL-cholesterol, and both were measured once with the use of a homogeneous enzymatic test (serum sample) based on color as modified by Roche. All measures were z-transformed and a mean score was calculated. 

#### 2.4.12. Subjective Health and Health Complaints

Subjective health, psychosomatic complaints, pains with regard to the locomotor system, as well as ear, eye, and throat complaints, which were combined to form one set of complaints, are assessed. Subjective health is described by one item asking participants to rate their subjective health on a 5-point scale from 1 (*very good*) to 5 (*very bad*). The psychosomatic complaints parcel consists of headache, stomach ache, abdominal pain, backache, and chest pain. Three response options were given: “yes, repeatedly”, “yes, once”, and “no”. The pains with regard to the locomotor system parcel is described by arm pain and leg pain with the same rating options as for the psychosomatic complaints and the earache, eyestrain, and sore throat parcels. 

### 2.5. Research Design

At the examination visit, health questionnaires were completed by either the parents of children aged 4–10 years or, by the participants themselves for participants aged 11–17 years. The medical examination was conducted by a physician. The fitness examination was conducted by trained examination teams. The survey lasted two hours on average [26]. 

### 2.6. Data Analysis

For analyses, all measurements of children and adolescents were partialed out and thus controlled for gender and age. Path analyses and bivariate correlations were conducted with AMOS 20.0 using a maximum likelihood algorithm (FIML). Because of missing data, the assumption of multivariate normality could not be tested. In addition to the *χ*^2^ test, we also used fit indices for model evaluation. The assessment of the global goodness-of-fit was based on the Root Mean Square Error of Approximation (RMSEA), as recommended by Hu and Bentler [40], and, additionally, on the Comparative Fit Index (CFI), as recommended by Beauducel and Wittmann [41]. According to Hu and Bentler [40], cut off values of approximately RMSEA ≤ 0.06 and CFI ≥ 0.95 are appropriate. Missing data were imputed with the full information maximum likelihood algorithm [42]. Even when the assumption of multivariate normality is violated, FIML provides relatively good estimations compared to deletion or mean imputation methods [43]. In cases where the results suggest mediation, moderation or suppression effects, they were tested only for the relevant variables in AMOS afterwards. For moderation effects, the product term was first calculated in SPSS 20.0. Moderation analyses were then conducted in AMOS. 

## 3. Results

### 3.1. Bivariate Path Correlations and Model Fit

Table 1 provides bivariate path correlations to ensure that previously identified relationships also exist in the present dataset. The biopsychosocial model (see Figure 1) revealed an acceptable degree of overall model fit, *χ*^2^(62) = 1,028.38, *p* < 0.001, RMSEA = 0.059, 90% confidence interval: 0.056–0.062, CFI = 0.80. The CFI is often smaller when analysis is based on questionnaire data [41]. Furthermore, to verify the loadings observed within the model, correlated error terms on the second and fifth level of the model as well as direct path on health and PF from SES were added. The CFI then improved to 0.90 and differences in the relevant path values of the model were close to zero or zero. 

Table 1Bivariate correlations, loadings, and explained variance.

**Table:** 

	1		2		3		4		5		6		R^2^
	a	r	a	r	a	r	a	r	a	r	a	r	
1 Socio-economic status	-												
2 Rural-urban differences	-	0.03 ^1^											
3 Immigration background	-	−0.25 ^3^	-	0.18 ^3^									
4 Intrinsic motivation	−0.09 ^3^	−0.06 ^3^	−0.02	−0.02	−0.01	−0.01							2.3
5 Physical activity of relatives and peers	0.24 ^3^	0.25 ^3^	−0.02	−0.01	−0.03	−0.09 ^3^	0.14 ^3^	0.12 ^3^					6.3
6 Quality of life	0.04	0.06 ^2^	−0.01	−0.02	−0.10 ^3^	−00.10 ^3^	0.20 ^3^	0.23 ^3^	-	-			1.2
7 Psychopathological problems	−0.14 ^3^	−0.15 ^3^	0.02	0.03	0.04 ^1^	0.08 ^3^	−0.08 ^3^	−0.09 ^3^	-	-	−0.59 ^3^	−0.60 ^3^	2.6

**Table:** 

	1		2		3		4		5		6		7		8		9		R^2^
	a	r	a	r	a	r	a	r	a	r	a	r	a	r	a	r	a	r	
8 Eating pattern	−0.12 ^3^	−0.14 ^3^	−0.05 ^3^	−0.05 ^3^	0.04 ^1^	0.06 ^3^	-	-	-	-				0.06 ^2^					2.5
9 Physical activity	0.05 ^3^	0.12 ^3^	−0.01	−0.02	−0.03	−0.08 ^3^	0.17 ^3^	0.22 ^3^	0.25 ^3^	0.30 ^3^	0.09 ^3^	0.14 ^3^	0.03	−0.07 ^2^	0.05 ^1^	0.03 ^1^			12.6
10 Sedentary behavior	−0.14 ^3^	−0.22 ^3^	0.05 ^2^	0.05 ^2^	0.05 ^1^	0.12 ^3 ^	-	-	−0.10 ^3^	−0.14 ^3^	−0.06	−0.05	−0.04	0.02	0.15 ^3^	0.15 ^3^	−0.02	0.08 ^3^	6.6

**Table:** 

	8		9		10		11			12		13		R2
	a	r	a	r	a	r	a	r		a	r	a	r	
11 Physical fitness	0.09 ^3^	0.10 ^3^	0.24 ^3^	0.25 ^3^	−0.10 ^3^	−0.10 ^3^								8.0
12 Objective health	−0.10^3^	−0.10 ^3^	−0.01	−0.09 ^3^	0.08 ^3^	0.09 ^3^	−0.29 ^3^		−0.31 ^3^					11.0
13 Subjective health	-	-	-	-	-	-	−0.15^3^		−0.17 ^3^	0.06 ^1^	0.11 ^3^			3.1
14 Psychosomatic complaints	-	-	-	-	-	-	−0.11^3^		−0.11 ^3^	0.01	0.05 ^1^	0.21 ^3^	0.26 ^3^	1.2
15 Pains with regard to the locomotor system	-	-	-	-	-	-	−0.02		0.00	−0.02	−0.03	0.07 ^2^	0.13 ^3^	0.2
16 Earache, eyestrain, and sore throat	-	-	-	-	-	-	0.04		0.03	0.02	0.00	−0.07 ^1^	−0.17 ^3^	0.1

a = paths of the sets; r = bivariate correlations; R^2^ = explained variance; ^1^ *p* < 0.05; ^2^ *p* < 0.01; ^3^ *p* < 0.001.

### 3.2. Loadings

#### 3.2.1. Level 1: Immigration Background, Rural-Urban Differences, and SES

Lower SES was observed in urban areas and for children and adolescents with parents with migration background. Further, children and adolescents with parents with migration background were more frequently seen to be living in urban environments (see Table 1). Higher SES was associated with more PA of relatives and peers, less intrinsic motivation, fewer psychopathological problems, better eating patterns, more PA, and less SB. The direct path of SES to QOL was not significant. Rural-urban differences showed no significant correlation with PA of relatives and peers, intrinsic motivation, QOL, psychopathological problems, and PA. Children and adolescents living in an urban environment revealed better eating patterns and more SB. Immigration background was associated with lower QOL, more psychopathological problems, poorer eating patterns and more SB. The direct paths of immigration background to PA of relatives and peers, intrinsic motivation, and PA were not significant. 

#### 3.2.2. Level 2: Psychopathological Problems, QOL, Intrinsic Motivation, and Physical Activity of Relatives and Peers

A more active environment was associated with a higher intrinsic motivation, less SB and more PA. More intrinsic motivation was associated with more PA, higher QOL, and fewer psychopathological problems. A higher QOL was associated with fewer psychopathological problems, more PA, but not with SB and eating patterns. The direct paths of psychopathological problems to SB, PA, and eating patterns were not significant. 

#### 3.2.3. Level 3: SB, PA, and Eating Patterns

Better eating patterns were associated with less PA, less PF, and poorer objective health. PA showed neither a significant correlation to SB nor to objective health. However, more PA was associated with better PF. Less SB was associated with higher levels of PF and better objective health.

#### 3.2.4. Level 4: Objective Health and PF

Better PF was associated with better objective health, better subjective health, and fewer psychosomatic complaints. The direct relationships of PF to pains with regard to the locomotor system, and earache, eyestrain, and sore throat, were not significant. Better objective health correlated with better subjective health but not with pains with regard to the locomotor system or psychosomatic complaints. 

#### 3.2.5. Level 5: Subjective Health and Health Complaints

Subjective health was correlated with all three sets of complaints. Whereas fewer psychosomatic complaints and lower pains with regard to the locomotor system were associated with poorer subjective health, lower earache, eyestrain, and sore throat was associated with better subjective health. 

## 4. Discussion

There is evidence that unhealthy behaviors are one of the major causes of chronic diseases and increased health care costs. Therefore, there is also a consensus that promoting good/positive health behavior would promote health and decrease health care costs [44]. The aim of this paper was to get information about what is needed to promote health behaviors, and consequently to promote objective health by identifying the interplay of distal and proximal factors of health behaviors and their consequences on objective health within a comprehensive approach: a biopsychosocial model was used to simultaneously examine relationships between environmental, individual, and behavioral factors of objective health and subjective health and health complaints. Results provide support for the biopsychosocial model and thus for comprehensive approaches to (subjective) health. Findings within the biopsychosocial model were in consensus with many recent bivariate findings, but also revealed moderation, mediation and effects of common variance. Moreover, in some cases they shed more light on relationships which, to date, had not yet been the subject of much investigation. However, some results contradicted earlier findings (mainly with regard to eating patterns). Nevertheless, influences on all five levels emerged. These findings provide evidence for the complexity of objective health and subjective health, and health complaints.

### 4.1. Key Findings of the Model Paths

#### 4.1.1. Level 1: SES

As expected, a higher SES was strongly (compared to other path values of the present analyses) associated with a more active environment [17,45]. A higher SES was also associated but to a smaller extent with a lower intrinsic motivation of children and adolescents to be physically active (Public authorities for social affairs, the family, and consumer protection, 2006), a higher level of PA itself [17], fewer psychopathological problems [46], and better eating patterns [18,47]; see Table 2 for an overview of studies for the present findings). Unexpectedly, we found no correlation between SES and QOL [15,16] and also small effects of common variance for intrinsic motivation to be physically active and psychopathological problems. It should be noted that the effect of SES on PA is quite smaller for path analyses compared to bivariate findings: the sole effect (controlled for the other distal and proximal factors) is smaller than that suggested by recent bivariate findings. Reanalyses (hypothesis-based) of these explorative findings are needed to shed more light on these findings. In a rather unexplored research area [48], we found a lower SB for a higher SES. Again this effect is lower when controlling for the other distal and proximal factors. Thus, enhancing the assumed preconditions of health behavior on the second level as well as the health behaviors themselves should be ensured with regard to SES. 

**Table 2:** Overview of studies for model path assumptions and findings.

Paths		Previous research		Findings of the present study	
		Authors	Main results	Path model	Comparison of bivariate and path model findings ^1^
Socio-economic status, rural-urban differences		Ferriss (2006) [ 49]	Interplays of socio-economic status, rural-urban differences, immigration status	Lower socioeconomic status was associated with immigration background and urban-dwelling children, and adolescents. Children and adolescents with parents with migration background live in urban areas more frequently.	
	Health	Islam *et al.* (2008) [50] Steffen *et al.* (2006) [51]	Connection to health outcomes	Indirect effects on health outcomes	
Socio-economic status &	Physical activity (of relatives and peers )	Hanson *et al.* (2007) [17] Kamphuis *et al.* (2007) [45]	Less participation in physical activity for lower socioeconomic status	In accordance	
	Intrinsic motivation	Public authorities for social affairs, the family, and consumer protection (2006) [52]	A higher intrinsic motivation to be physically active for lower socio-economic status	In accordance	
	Quality of life	Ravens-Sieberer *et al.* (2008) [16]	Negative impact of lower socio-economic status on quality of life	No differences in quality of life by socio-economic status	Probably effects of common variance for intrinsic motivation & psychopathological problems
	Psychopathological problems	Sellstrom & Bremberg, (2006) [46]	Children living in deprived areas display psychopathological problems more frequently	In accordance	
	Eating patterns	Morland *et al.* (2002) [18] Eikenberry & Smith, (2004) [47]	Poorer eating patterns for lower socio-economic status	In accordance	
	Sedentary behavior	Whitt-Glover *et al.* (2009) [48]	Some evidence for lower sedentary behavior with a higher socio-economic status	Lower sedentary behavior for a higher socio-economic status	
		Hanson & Chen, (2007) [ 17]	Inconsistent findings; more recent research has suggested lower levels of physical activity for lower socio-economic status	Lower levels of physical activity for lower socio-economic status	
Rural-urban differences &	Physical activity (of relatives and peers )	Trost *et al.* (2002) [53]	Higher levels of leisure time physical activity among adults living in rural areas than for adults living in urban areas	No differences	
	Intrinsic motivation	Monge-Rojas *et al.* (2009) [54]	Scant information available; similar emergent themes of barriers and motivators for achieving an active lifestyle for rural and urban Costa Rican adolescents	No differences	
	Quality of life	Collier *et al.* (2000) [55] Camfield & Ruta (2007) [56]	Scant information available; no rural-urban differences in quality of life in a developed country; but seen in developing countries	No differences	
	Psychopathological problems	Roussos *et al.* (2001) [57] Heyerdal *et al.* (2004) [58] Nie *et al.* (2008) [59]	Inconsistent findings on rural-urban differences and psychopathological problems may be due to culture as well as to different operationalizations	No differences	
	Eating patterns	Adair & Popkin (2005) [ 60]	Scant information available; in China, Russia, Cebu, the Philippines, and the United States, higher percentages of snacking and of calories were found for children and adolescents living in urban environments	Poorer eating patterns for rural children and adolescents	
	Sedentary behavior	Biddle (2007) [ 61] Li *et al.* (2007) [62] Shi *et al.* (2006) [63] Springer *et al.* (2006) [64]	Scant and inconsistent information available on differences in sedentary behavior caused by rural-urban differences	Children and adolescents living in urban environments revealed more sedentary behavior	
	Physical activity	Sjolie & Thuen (2002) [ 65] Nelson, Gordon-Larsen, Song, & Popkin (2006) [66] Yamamoto-Kimura *et al.* (2006) [67]	Inconsistent findings on rural-urban differences and physical activity with regard to nationality, age, and measurement method	No differences	
Immigration background &	Physical activity (of relatives and peers )	Dotevall *et al.* (2000) [68] Lindström & Sundquist (2001) [69] Green *et al.* (2003) [70]	Higher prevalence of physical inactivity for certain groups of children and adolescents with parents with migration background , also associated with socioeconomic differences; research on of children and adolescents with parents with migration background and physical activity levels has been rare	No differences	Rural-urban differences was shown to be a moderator, indicating common variance in the association of immigration background on physical activity of relatives and peers; quality of life, and physical activity of relatives and peers were found to be mediators; common variance with sedentary behavior and eating patterns could not be excluded
	Intrinsic motivation	Hosper *et al.* (2008) [71]	Scant Information on the correlation of immigration background and intrinsic motivation to be physically active	No correlation	
	Quality of life	Pantzer *et al.* (2006) [72]	Effects on quality of life of children’s immigration experience have been examined only marginally; most of these studies have focused on specific aspects such as psychological well-being, mental health, and self-esteem	Children and adolescents with parents with migration background showed a lower quality of life compared to native children and adolescents	
	Psychopathological problems	Fazel & Stein (2003) [ 73] Leavy *et al.* (2004) [74]	Studies with children with parents with migration background have revealed more psychopathological problems compared to ethnic minority children	In accordance, children and adolescents with parents with migration background revealed more psychopathological problems	A moderation effect was shown for socio-economic status; assumption of common variance for quality of life and intrinsic motivation to be physically active
	Eating patterns	Kumar & Wandel (2006) [ 75]	Immigrants are confronted with nutritional problems	In accordance, poorer eating patterns were observed for children and adolescents with parents with migration background than for native children and adolescents	There seemed to be common variance effects for physical activity and sedentary behavior
	Sedentary behavior	Singh *et al.* (2008) [76] Allen *et al.* (2007) [77]	Inconsistent findings on the relationship between immigration background and sedentary behavior	Sedentary behavior in the present study was more widespread in children and adolescents with parents with migration background	Socio-economic status, rural-urban differences, psychopathological problems, and quality of life were moderators of the relationship between immigration background and sedentary behavior
Physical activity (of relatives and peers ) &	Intrinsic motivation	Lämmle *et al.* (2011) [78]	Positive relationship between the physical activity of relatives and peers and intrinsic motivation	In accordance	
	Physical activity	Lämmle *et al.* (2011) [78]	Higher levels of activity of relatives and peers were associated with higher levels of activity for children and adolescents	In accordance	
	Sedentary behavior	Saelens & Kerr (2008) [ 79]	The parents’ sedentary behavior was previously shown to be positively associated with their children’s sedentary behavior; research gap on hypothesis as to whether or not a physically active environment also positively affects the sedentary behavior of children and adolescents in the direction of less sedentary behavior	Sedentary behavior was lower for higher levels of activity of relatives and peers	
Intrinsic motivation &	Quality of life, psychopathological problems	Weiner (1986) [ 80] Lustyk *et al.* (2004) [81]	Referring to attribution theory, it can be assumed that intrinsic motivation as a reason for engaging in PA could affect psychological outcomes such as quality of life and psychopathological problems; associations between exercise motivation and quality of life	In accordance; results revealed fewer psychopathological problems and higher quality of life for a higher intrinsic motivation to be physically active	
	PA	Woods *et al.* (2007) [82]	Intrinsic motivation was shown to be predictive of PA in children and adolescents	In accordance	
Quality of life &	Psychopathological problems	Ravens-Sieberer *et al.* (2008) [15]	Higher quality of life rates in children and adolescents in 21 European countries (among them Germany) were associated with more psychopathological problems	In accordance	
	Sedentary behavior	Lee *et al.* (2009) [83]	Initial findings with quality of life factors have suggested that parental limits and family conflict are predictive of sedentary behavior; it was hypothesized that a higher quality of life would lead to a reduced sedentary behavior pattern	No correlation	
	Physical activity	Sánchez-López *et al.* (2009) [84]	Scant information on the relationship of quality of life and physical activity; higher levels of physical activity have been associated with higher quality of life	In accordance	
	Eating patterns	Wang *et al.* (2008) [10] Chen *et al.* (2005) [85]	Scant information on the relationship of quality of life and eating patterns in childhood and adolescence; unhealthy eating patterns are a risk factor for poor quality of life	No correlation	
Psychopathological problems &	Sedentary behavior	Griffith *et al.* (2007) [86] Hamer *et al.* (2009) [87]	Inconsistent findings on the association between psychopathological problems and sedentary behavior	The assumption that more psychopathological problems would lead to more sedentary behavior was not confirmed.	
	Physical activity	Wiles *et al.* (2008) [88]	Reduced psychopathological problems when a physically active lifestyle is maintained	No correlation	Mediation effect was observed for intrinsic motivation to be physically active; common variance effects for eating patterns and sedentary behavior could not be excluded
	Eating patterns	Mamun *et al.* (2009) [89]	Eating patterns are often seen as being confounded or as mediating factors for the association between psychopathological problems and obesity (or being overweight) in children and adolescents	No correlation	There could be common variance between physical activity and sedentary behavior.
Eating patterns &	Physical activity, sedentary behavior	Anderson & Butcher (2006); [ 90] Sallis *et al.* (2009) [19]	Eating patterns, physical activity, and sedentary behavior have been viewed as the primary modifiable behaviors of energy imbalance. Higher physical activity levels have been shown to be related to a healthy diet.	Unhealthy dietary patterns were associated with higher levels of physical activity and physical fitness as well as with better health	
	Sedentary behavior	Kutchman *et al.* (2009) [91] Kremers *et al.* (2007) [92]	Sedentary behavior is positively associated with the consumption of energy-dense snacks and sugar-containing drinks	In accordance; unhealthier food intake occurred more when sedentary behavior was higher	
	Physical fitness	Brunet *et al.* (2007) [93]	As two other well-known correlates of eating patterns, obesity and being overweight are associated with lower physical fitness levels		
	Health	Lindadakis *et al.* (2008) [94]	Inverse associations of physical fitness and eating patterns with metabolic syndrome risk factors. Unhealthier eating patterns are associated with obesity, being overweight		
Physical activity &	Sedentary behavior	Sallis *et al.* (2009) [19]	Weak association between physical activity and sedentary behavior;		
		Hills *et al.* (2007) [2]	physically active children and adolescents have exhibited sedentary behavior, even on the same day	In accordance	Common variance was present for socioeconomic status, rural-urban differences, immigration background, physical activity of relatives and peers, quality of life, psychopathological problems, and eating patterns
	Physical fitness	Castelli & Valley (2007) [ 95]	Regular engagement in physical activity has been shown to be associated with the attainment of standards addressing physical fitness	In accordance; higher levels of physical activity had an enhancing effect on physical fitness	
	Health	Sallis *et al.* (2009) [19]	Higher levels of physical activity were associated with better health	No correlation	Mediation effect of physical fitness; common variance of sedentary behavior and eating patterns cannot be excluded;
Sedentary behavior &	Physical fitness	Kerner (2005) [ 96]	The relationship between sedentary behavior and physical fitness has been hypothesized more than it has been explored	Our underlying assumption that less sedentary behavior would be associated with higher levels of physical fitness was confirmed	
	Health	Sallis *et al.* (2009) [19]	Sedentary behavior is contributing to obesity since it is characterized by low energy expenditure	In accordance; we found a negative association of sedentary behavior with health	
Physical fitness &	Health, subjective health, psychosomatic complaints, pains with regard to the locomotor system, earache, eyestrain, and sore throat	Eiberg *et al.* (2005) [97]	There has been some evidence that PF is related to health and subjective health in children	Better health, better subjective health, and fewer psychosomatic complaints revealed for higher physical fitness levels. No significance emerged for pains with regard to the locomotor system and earache, eyestrain, and sore throat.	
Health &	Subjective health, psychosomatic complaints, pains with regard to the locomotor system, earache, eyestrain, and sore throat	Janssen *et al.* (2004) [20]	Health-relevant parameters have been shown to be associated with physical and psychological health complaints	Health was associated with subjective health, but not with pains with regard to the locomotor system, psychosomatic complaints and earache, eyestrain, and sore throat	The diminishing effect of health on psychosomatic complaints within the biopsychosocial process model seemed to be due to collinearity with physical fitness
Subjective health &	Psychosomatic complaints, pains with regard to the locomotor system, earache, eyestrain, and sore throat	Vetter (2007) [ 98]	Self-reported occurrence of pain and/or illness influenced the general perception of subjective health	Poorer subjective health was associated with fewer psychosomatic complaints and lower, pains with regard to the locomotor system, but higher earache, eyestrain, and sore throat	For the relationships between subjective health and pains with regard to the locomotor system and between subjective health and earache, eyestrain, and sore throat common variance shared with physical fitness and health emerged

^1^ Moderating, mediating and suppression effects were examined.

#### 4.1.2. Level 1: Rural-Urban Differences

In general there has been a lack of research on rural-urban differences and (factors affecting) health behavior (e.g., [61]). Most of the correlations assumed and analyzed here were not significant for German children and adolescents. Small rural-urban differences were only found for the two health behaviors of eating patterns [60] and SB [61,62,63,64], with poorer eating patterns for rural-living children and adolescents and more SB for urban-living children and adolescents. The prevention focus with regard to rural-urban differences for German children and adolescents should therefore lie on eating patterns and SB. 

#### 4.1.3. Level 1: Immigration Background

Filling research gaps, no difference was observed for immigration background and intrinsic motivation for being physically active [71]. However, even though the effects were small, for children and adolescents with parents with migration background, lower QOL [72] as well as more psychopathological problems [73,74,76,77] emerged in the present data. Unexpectedly, we found common variance between immigration background and rural-urban differences in the association with PA of relatives and peers. Furthermore, we found mediation effects of QOL, and PA of relatives and peers on the relationship of immigration background and PA. Expectedly, children and adolescents with parents with migration background had poorer eating patterns than natives (small effect) [75]. However, there were also unexpected effects of common variance. Common variance and moderation effects were also found for the relationship between immigration background and psychopathological problems as well as immigration background and SB. In both cases SES was a significant moderator indicating that the relationship between immigration background with psychopathological problems and the relationship between immigration background and SB is stronger for a lower SES. All moderation effects were nearly equivalent. In sum, we rarely found a sole effect of immigration background. Thus, further biopsychosocial approaches are needed to replicate these findings. If findings could be replicated, information on how exactly to promote health (determining) behaviors for children and adolescents with parents with migration background (e.g., PA of relatives and peers or QOL should also be considered) would be available. 

#### 4.1.4. Level 2: PA of Relatives and Peers

Expectedly, the PA of relatives and peers was positively associated with the intrinsic motivation of being physically active and even to a higher extent with PA itself [78]. Filling a research gap, we found that the PA of relatives and peers was also associated with SB in a small but positive sense. Thus, the PA level of relatives and peers seemed to be important to the child’s movement behavior. 

#### 4.1.5. Level 2: Intrinsic Motivation to be Physically Active

Again filling a research gap, we found that the intrinsic motivation to be physically active was strongly associated with QOL [81] and psychopathological problems (small effect). At the same time, it expectedly was moderately associated with a higher PA [82], emphasizing the relevance of these three factors on the second level of the biopsychosocial model. 

#### 4.1.6. Level 2: QOL

As expected, QOL and psychopathological problems were strongly inversely related [15,16]. Research on the association of QOL with health behavior has been rare and QOL is frequently seen to be related to behavior [84,85,99]. However, our research focus was oriented towards influences on health-relevant behaviors. We therefore viewed behaviors as an effect of QOL. What we found was that only PA was slightly positively associated. 

#### 4.1.7. Level 2: Psychopathological Problems

Unexpectedly, we found no association between psychopathological problems and SB [87,100]. Again unexpectedly, we found mediation effects of intrinsic motivation to be physically active for the small correlation between psychopathological problems and PA [88]. As with psychopathological problems and SB, we found no association between psychopathological problems and eating patterns [89]. 

#### 4.1.8. Level 3: Eating Patterns

Even though the effects were small, we found totally unexpectedly that unhealthier eating patterns were observed for more active children and adolescents [93] and those with a better PF [19]. Furthermore, unhealthier eating patterns were associated with better objective health [91,94]. In the current study, there was no evidence for a mediation effect of PA and PF on eating patterns and objective health as was demonstrated in the biopsychosocial model tested earlier [78]. Moderating effects of PA and PF could have an interesting interventional aspect since energy expenditure plays an important role in the occurrence and consequences of obesity and of being overweight [20]. It would appear that PA and PF can buffer inadequate energy intake if both are positively related to objective health. A possible explanation of this unexpected finding might be that remembering the complete consumption of the past weeks might be difficult for parents as well as for children (from 4–10 years of age) and adolescents (from 11–17 years of age). As expected, we found a PF-enhancing effect of PA [95]. 

#### 4.1.9. Level 3: PA and SB

Unexpectedly but plausibly, we found that the effect of PA on objective health [19] was mediated by PF, emphasizing the meaning of PF in objective health research in childhood and adolescence. Again unexpectedly, effects of common variance emerged for the correlation of PA and SB. Filling a research gap we observed that higher SB was associated with lower PF [96]. Expectedly, we found better objective health for those with lower SB [19]. Both results indicated the relevance of SB in health research, even though the effects were small. 

#### 4.1.10. Level 4: PF and Objective Health

As expected [97], PF not only led to better objective health (strong effect) but also to a better subjective valuation of objective health in children and adolescents. Compared to PF, objective health seemed to be less meaningful for the subjective rating of objective health status in childhood and adolescence [20], again emphasizing the significance of PF in health research.

#### 4.1.11. Level 5: Subjective Health and Health Complaints

Information about the interplay of different subjective health ratings was lacking; thus, the current findings should be a basis for future research. However, we did not expect the findings of fewer psychosomatic complaints and lower pains with regard to the locomotor system with poorer subjective health. Maybe children and adolescents refer to parameters other than psychosomatic complaints (strong effect) and pains with regard to the locomotor system (small effect) when rating their globally perceived subjective health. Thus, results suggest that a more differentiated measurement of subjective health is necessary. 

### 4.2. Limitations

Proximal intrapersonal factors such as self-efficacy, which has been shown to be closely related to PA, or interpersonal factors such as social support and social interaction as important predictors for morbidity and mortality [101] were not assessed. However, this study found, first of all, support for comprehensive approaches based on variables chosen from experts of several disciplines. These explorative findings should be replicated and future approaches should expand this research further or take other determinants into account. Furthermore, this is a cross-sectional study so causality cannot be examined. Consequently, longitudinal research on the association of QOL with psychopathological problems on health behavior is needed. It would be valuable in the future to also take into account eating patterns and SB of relatives and peers, as it can be shown that the PA of relatives and peers is related to PA and SB in children and adolescents. Finally, we did not analyze the model for different ages and genders. Nevertheless, the samples of each age group were too small. Further research on different relevant age groups could yield highly relevant results. Besides, it would be interesting to examine whether gender differences also occur in biopsychosocial analyses. However, the focus of the present paper was a first attempt to simultaneously analyze a five level model with several biopsychosocial factors of health behavior and objective health. After first finding support for such comprehensive approaches, future research should now also pay respect to age and gender differences in this field of research. One might criticize that some of the path values are low (even though significant). The interpretation of the effect size within the discussion part was oriented on the range of the model path values in order to see which associations seemed to be more fruitful than others for prevention or intervention measures. Another interpretation possibility would have been to discuss the cost-efficiency effect for prevention or intervention measures; e.g., what are the health care costs of lower fitness and health in children and adolescents compared to the benefit of smaller prevention or intervention measures. 

With regard to study and design, it should be noted that neither items to assess intrinsic motivation nor SB have been validated yet. Also, more items (e.g., time spent with mobile phone, computer, and homework) should be used to assess SB in its entirety in future analyses. Additionally, future analyses might differentiate between different cultures when considering immigration background since different cultures of children and adolescents with parents with migration background might have different implications for factors affecting health behaviors. 

## 5. Conclusions

In summary, we analyzed several distal and proximal determinants of health behaviors and their associations to (subjective) health simultaneously in this paper in an attempt to shed light on this complex interplay of factors. With the use of a 5-level model, support for comprehensive approaches and detailed information on (subjective) health-affecting, biopsychosocial factors in children and adolescents was shown. Results led to the assumption of a number of suppression, moderation, and mediation effects within the biopsychosocial model. These findings also emphasize the importance of comprehensive compared to bivariate analyses in health research: In addition to the results we had expected, these unexpected findings (e.g., the effect of PA on objective health was mediated by PF) may provide directions for future research. Moreover, some initial research (e.g., with regard to immigration background) was included on the biopsychosocial model, and again, directions for future research can be derived from these findings. Replication of unexpected and initial findings would be significant for future preventive measures (e.g., enhancing the physical activity of relatives and peers for children with a lower SES is associated with an enhancement of PA of children and adolescents, which leads to better PF and health; enhancing the QOL of children with immigration background is also associated with an enhancement of PA in children and adolescents, which leads to better PF and health). 
